# Acute phase protein response in an experimental model of ovine caseous lymphadenitis

**DOI:** 10.1186/1746-6148-3-35

**Published:** 2007-12-19

**Authors:** Peter D Eckersall, Fraser P Lawson, Laura Bence, Mary M Waterston, Tamara L Lang, William Donachie, Michael C Fontaine

**Affiliations:** 1 Division of Animal Production and Public Health, Faculty of Veterinary Medicine, University of Glasgow, Bearsden Rd, Glasgow, G61 1QH, UK; 2 Moredun Research Institute, International Research Centre, Pentlands Science Park, Bush Loan, Penicuik EH26 0PZ, UK

## Abstract

**Background:**

Caseous lymphadenitis (CLA) is a disease of small ruminants caused by *Corynebacterium pseudotuberculosis*. The pathogenesis of CLA is a slow process, and produces a chronic rather than an acute disease state. Acute phase proteins (APP) such as haptoglobin (Hp) serum amyloid A (SAA) and α_1 _acid glycoprotein (AGP) are produced by the liver and released into the circulation in response to pro-inflammatory cytokines. The concentration of Hp in serum increases in experimental CLA but it is not known if SAA and AGP respond in parallel or have differing response profiles.

**Results:**

The concentration in serum of Hp, SAA and AGP in 6 sheep challenged with 2 × 10^5 ^cells of *C. pseudotuberculosis *showed significant increases (P < 0.05) compared to 3 unchallenged control sheep. By day 7 post infection. (p.i.) the Hp and SAA concentrations reached mean (± SEM) values of 1.65 ± 0.21 g/L and 18.1 ± 5.2 mg/L respectively. Thereafter, their concentrations fell with no significant difference to those of the control sheep by day 18 p.i.. In contrast, the serum AGP concentration in infected sheep continued to rise to a peak of 0.38 ± 0.05 g/L on day 13 p.i., after which a slow decline occurred, although the mean concentration remained significantly higher (P < 0.05) than the control group up to 29 days p.i.. Specific IgG to phospholidase D of *C. pseudotuberculosis *became detectable at 11 days p.i. and continued to rise throughout the experiment.

**Conclusion:**

The serum concentrations of Hp, SAA and AGP were raised in sheep in an experimental model of CLA. An extended response was found for AGP which occurred at a point when the infection was likely to have been transforming from an acute to a chronic phase. The results suggest that AGP could have a role as a marker for chronic conditions in sheep.

## Background

Caseous Lymphadenitis (CLA) is a chronic disease of sheep and goats caused by *Corynebacterium pseudotuberculosis *and is characterised by the formation of pyogranulomas within the superficial lymph nodes draining the site of infection. Subsequent dissemination of this facultatively intracellular organism via the lymphatic or blood systems can also result in the formation of similar abscesses within the internal organs and other internal and external lymph nodes, most frequently the mediastinal lymph node and lungs [1,2]. As lesions progress they become encased within fibrous capsules, the inflammatory immune response decreases, and continued slow expansion of the abscess may then occur [3]. It is known that cytokines are involved in the host response to infection, with inflammatory cytokines (such as tumour necrosis factor-α (TNF-α), interleukin (IL) 1-β and IL-6) being expressed at the site of inoculation and T-cell associated cytokines (IL-2, IL-4 and interferon-γ (IFN-γ)) being expressed at lymph nodes [4,5]. Indeed measurement of the production of IFN-γ by peripheral blood lymphocytes in response to *C. pseudoturberculosis *antigen has been reported as a marker of cell-mediated immunity in CLA [2]. Inflammatory cytokines such as TNF-α, IL-1β and IL-6 are known to stimulate the systemic acute phase response [6] and this pathophysiological reaction has indeed been identified in experimental CLA. Increases in serum α and β protein fractions have been reported [7] and these fractions contain many acute phase proteins. An increase in concentrations of serum haptoglobin (Hp), a major acute phase protein in sheep and other ruminants [8], has also been reported in response to *C. pseudotuberculosis *infection [9].

The acute phase proteins are a group of serum proteins which are produced and released by the liver following stimulation by pro-inflammatory cytokines such as IL-6 and TNF-α [10,11]. In ruminants, haptoglobin (Hp) and serum amyloid A (SAA) are known to be major acute phase proteins increasing up to 1,000-fold on stimulation, while α_1 _acid glycoprotein (AGP) is a moderate acute phase protein which can increase several fold from its normal plasma concentration [12,13]. Although called the acute phase proteins their presence in the circulation can persist beyond the immediate post-infection time period, although, as pathological lesions become chronic, the pattern of response can vary. In a study of cases involving acute and chronic inflammatory reactions in cattle, increases in SAA and Hp were associated with acute conditions while increased AGP was more common in chronic inflammatory conditions [14]. The acute phase proteins are integral to the non-specific innate immune response and can be a valuable quantitative marker of the level of pathogenesis in experimental studies of disease as well as being useful as biomarkers of natural infection [15-17].

This study had the objective of evaluating the acute phase protein response of SAA and AGP in relation to that of Hp during experimentally-induced *C. pseudotuberculosis *infection, in order to determine their potential value in monitoring the progress of the disease. Furthermore, the relation of the APP to the development of acquired immunity, as determined by measuring the increase in serum IgG antibody to the *C. pseudotuberculosis *phopspholipase D (PLD) protein, was investigated.

## Results

### Experimental model of CLA

The experimental model of CLA used in this study has been reported elsewhere [18]. In the previous study it was demonstrated that subcutaneous inoculation of sheep with 10^4 ^colony-forming units of a virulent, UK *C. pseudotuberculosis *isolate was sufficient to reproduce the symptoms observed in naturally-occurring CLA. In the current study, lesion development was initially observed at the local-drainage lymph node, following which dissemination of infection lead to the affectation of further, random lymph nodes. Approximately 25% of animals were also observed to develop lung lesions, which is consistent with infection in the field. To ensure a 100% rate of infection due to the inclusion of a relatively small number of study animals, 2 × 10^5 ^*C. pseudotuberculosis *cells were administered instead of 10^4^; however, this dose was in no way expected to significantly alter the outcome of infection, based on an interpolation of the results of the previous study [18].

### Acute phase protein response in CLA

The acute phase response was determined by measurement of the Hp, SAA and AGP concentrations in serum from the *C. pseudotuberculosis*-infected animals.

#### Haptoglobin

**Figure 1:**
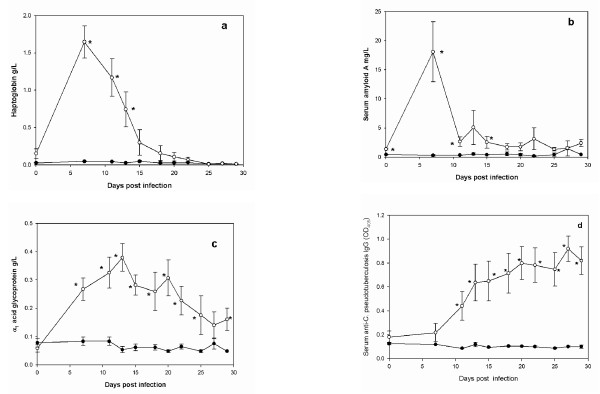
**Acute Phase Protein responses in experimental model of *C. pseudotuberculosis *infection**. Mean (± SEM) serum concentrations of Hp (a), SAA (b), AGP (c) and IgG (d) in 6 sheep infected with 2 × 10^5 ^cfu (open circle) and 3 unchallenged control sheep (closed circles). Asterisk (*) indicate points where the mean of concentration of the challenged sheep was significantly different (P < 0.05) from the mean of the unchallenged sheep.

The mean (± SEM) serum concentration of Hp was significantly (P < 0.05) raised at 7 days p.i. at 1.65 ± 0.21 g/L and remained significantly raised in comparison to the Hp concentration in serum from unchallenged sheep until 15 days p.i. (Figure 1a). The mean area under the curve (AUC) for the Hp response in infected animals was significantly greater (P < 0.05) than the AUC for control sheep (Table 1).

**Table 1:** Area under curves for the time course of acute phase protein and anti-PLD (IgG) following *C. pseudotuberculosis *infection

	**Haptoglobin** **Days.g/L** **Mean ± SEM**	**SAA** **Days.mg/L** **Mean ± SEM**	**AGP** **Days.g/L** **Mean ± SEM**	**IgG** **Days.OD_450_** **Mean ± SEM**
**Control group n = 3**	0.99 ± 0.12	2.67 ± 0.23	2.14 ± 0.24	3.56 ± 0.09
**Infected group n = 6**	16.28 ± 2.59	8.57 ± 1.40	7.68 ± 1.45	20.08 ± 3.26
*P	<0.05	<0.05	<0.05	<0.05

*for difference between control and infected groups by Mann-Whitney test

#### Serum amyloid A

The mean serum concentration of SAA was raised at 7 days p.i. at 18.1 ± 5.2 mg/L being significantly greater than the concentration in the control sheep (P < 0.05) and fell to 2.6 ± 1.0 mg/L by day 15 p.i. (Figure 1b). The mean AUC for the SAA response in infected animals was significantly greater (P < 0.05) than the AUC for control sheep (Table 1).

#### α_1 _Acid glycoprotein

The mean serum concentration of AGP showed a significant (P < 0.05) increase by day 7 p.i. but, in contrast to Hp and SAA, then showed a gradual increase with the mean concentration reaching 0.38 ± 0.05 g/L on day 13 p.i. (Figure 1c). Thereafter, the mean AGP concentration fluctuated with a gradual decline but the AGP concentration in samples from the infected sheep was still significantly different (P < 0.05) from the unchallenged group at the end of the experimental study period at day 29 p.i.. The mean AUC for the AGP response in infected animals was significantly greater (P < 0.05) than the AUC for control sheep (Table 1).

### Specific antibody (IgG) response

Serological analysis of the serum samples demonstrated a specific immune response to PLD, a conserved *C. pseudotuberculosis *virulence factor, which became significantly greater (P < 0.05) than the levels in the uninfected sheep at day 11 p.i. and continued to rise until the end of the study period at day 29 p.i. The mean AUC for the IgG response in infected animals was significantly greater (P < 0.05) than the AUC for control sheep (Table 1).

## Discussion

This investigation has confirmed that an APP response occurs in sheep experimentally-infected with *C. pseudotuberculosis*, and extends the previous report of an increase in the concentration of serum Hp in CLA [9] by demonstrating that increases also occur in the concentrations of SAA and AGP. The result of both the profile and AUC of the responses shows a significant increase in Hp, SAA and AGP in CLA. The profiles of the protein response of Hp and SAA were similar, although the fall in SAA after day 7 p.i. was more rapid than that of Hp. In contrast, the profile of the increase in the concentration of AGP was substantially different from the other APP with a more gradual increase followed by a slower decline in the serum concentrations of Hp and SAA. This difference in the profile of the APP during disease progression corresponds to previous studies in cattle, in which AGP was found to be elevated in a greater proportion of clinical cases involving chronic disease in comparison to conditions involving acute conditions [19]. The acute phase reaction of Hp in CLA has been described previously [9], but the reaction of SAA and the continued production of AGP during the transition to chronic stages of the infection are novel observations. The evidence here further supports the concept that raised AGP could be a biomarker of chronic conditions in sheep.

Most investigations of the acute phase protein response, and its control by pro-inflammatory cytokines, focus on the immediate or 'acute phase' response to the infection. CLA is known to develop as a chronic infection and the most interesting finding of the study is the extension of the AGP response into the later phase of the disease while the Hp and SAA responses fall more rapidly. The phases of infection during CLA have been described as an initial phase (day 1–4 p.i.) characterised by recruitment of neutrophils to the inoculation site and the draining lymph node, an amplification phase (day 5–10 p.i.) characterised by the development of pyrogranuloma and a stabilisation phase characterised by maturation and persistence of the pyrogranuloma [4]. On the basis of the previous study on Hp response [9] and with the results presented here, the initial and amplification phases would correspond to the acute phase of the infection with raised levels of Hp and SAA. The stabilisation phase, when Hp and SAA concentrations return towards the baseline while AGP is maintained at a significantly higher concentration than in controls would correspond to the period when the acute phase of CLA is transformed into a chronic condition.

The mechanism of the switch between acute phase and the chronic phase of infections has been a relatively neglected area of investigation, and the experimental CLA model used in this study would be a means for further investigation of this aspect of the innate immune system. It is possible that alterations in expression of cytokines at different stages of the infection could relate to the differing profile of APP responses. In a study of cytokine expression, following infection with wild type *C. pseudotuberculosis *it was found that at the site of inoculation, the inflammatory cytokines TNF-α and IL1-β were up regulated on day 7 p.i., whereas on day 28 p.i IL1-β and IL-6 showed increased expression while the expression of TNF-α had returned to baseline. In contrast, T-cell associated cytokines such as IL2, IL4 and IFN-γ were up regulated at the draining lymph node [4]. The contrasting AGP and Hp/SAA responses could be indicative of an alteration in the output of cytokine from these different sites.

Production of IFN-γ by leukocytes in response to excretory-secretory products of *C. pseudotuberculosis *was able to differentiate goats into high and low responding groups [5]. It would be of interest to determine if the variation found between individuals in their responses of Hp, SAA and AGP were also different between such groups. However the limited numbers in this study precluded assessment of such a relationship which would need larger experimental animal groups to provide a definitive answer. It could be possible that the use of the IFN-γ stimulation test for the presence of CLA [5] could be enhanced by simultaneous assessment of the Hp, SAA and AGP concentrations.

There has recently been growing evidence that the APP can be synthesised by non-hepatic tissue, such as lung [20], adipose[21] and mammary gland tissue [22], and activated neutrophils [23] and alveolar macrophages [24]; in the latter, differing AGP glycan isoforms were found during an acute phase response. It is possible that the extended acute phase response of AGP in serum in CLA could be a result of such non-hepatic synthesis of AGP, but it would require further investigation to assess this possibility and to identify the source of the increased production of this protein.

The increase in antibody titre against PLD was in agreement with previous studies of antibody response to experimental infection *with C. pseudotuberculosis *[18], though with other experimental approaches specific antibody can be demonstrated as early as 5 days after infection [5]. As would be expected with mediators of the innate and acquired immune systems, the APP responses were earlier than that of the specific antibody response to PLD. The concentrations of Hp and SAA were returning towards those in the control animals by the time the IgG levels were raised (day 11 p.i.) although the mean AGP concentration continued to rise after the IgG response was initiated and was consistent with a switch from the innate to the acquired immune responses. Of great interest would be an investigation to determine if the response of all or of any of the acute phase proteins in the immediate p.i. period, as measured by AUC, would correlate to the later IgG response in individual animals. The number of animals was not sufficient to examine this possibility in the present study.

The APP are valuable markers of disease in man and in animals [11,13] but in veterinary medicine are generally regarded as markers of the 'acute phase' of infection or immunity. The findings here demonstrate that AGP responds in different ways to the progress of infection compared to Hp and SAA and therefore the difference in response profile indicates that investigation of a range of APP could provide additional diagnostic information on the progress of disease. Feline AGP is known to be a differential test in the diagnosis of feline infectious peritonitis [25] and it could be that ovine AGP may serve the same purpose in the diagnosis and prognosis of CLA in sheep.

## Conclusion

An acute phase reaction which varied between APP has been identified in CLA. Haptoglobin and SAA increased in the post-infection period, returning to normal after 2 weeks while AGP increased more slowly and remained elevated for 4 weeks p.i.. These results indicate that the monitoring of a number of acute phase proteins can increase the diagnostic information available as a result of their analyses.

## Methods

### Experimental *C. pseudotuberculosis *infection model

For preparation of challenge inocula, the virulent, UK, ovine *C. pseudotuberculosis *isolate, 3/99-5 [26], was passaged three times at 37°C for 48 h on blood agar plates (Blood Agar Base (Oxoid; Basingstoke, Hampshire, UK) supplemented with 5% (v/v) sheep blood). Bacterial cells were harvested using sterile, cotton-tipped swabs and suspended in PBS in polystyrene Universal tubes. Cells/ml were initially estimated by use of a Densimat (bioMérieux, Basingstoke, UK), and then quantified accurately using a Thoma counting chamber. Subsequently, primary suspensions were diluted to obtain the required cells/ml for the infection experiment.

For experimental infection, 6 male, approximately 1-year-old Suffolk-cross sheep were challenged subcutaneously with a 1 ml inoculum, administered 2 cm caudal to the base of the left ear on a line passing through the ear to the left eye. The inoculum consisted of *ca*. 2 × 10^5 ^*C. pseudotuberculosis *cells, which was a level known from an earlier optimisation experiment to be sufficient to ensure a 100% infection rate and known to result in a clinical manifestation of disease identical to naturally observed CLA [18]. Subsequently, the group of infected animals was housed separately from a further 3 unchallenged control animals for the duration of the experiment. Blood was collected once in the first week of the experiment, and then three times weekly for the next 3 weeks for downstream analyses. The blood samples were allowed to coagulate, serum was removed after centrifugation at 1,730 × *g *for 20 min at 4°C and stored at -20°C prior to analysis for serum APP and the level of antibody to *C. pseudotuberculosis *PLD. Animal experiments were approved by the Moredun Research Institute's Experiments Committee and conducted by appropriately licensed personnel.

### Serology

The immunological response of each animal to challenge with *C. pseudotuberculosis *was determined by measurement of serum anti-PLD IgG by enzyme-linked immunosorbent assay (ELISA) using recombinant-PLD coated plates, as described elsewhere [18], with results expressed as the absorbance at 450 nm (OD_450_).

### Analysis of acute phase proteins

The concentration of Hp in serum samples was determined using a Hp-haemoglobin binding assay as described previously [27]. Serum SAA concentrations were determined by use of a commercial ELISA obtained from Tridelta Development plc. (Dublin, Ireland), according to the manufacturer's instructions, as previously described for bovine serum [22]. The concentration of AGP in sera was determined by radial immunodiffusion with a kit obtained from J-Path Inc. (Tokyo, Japan), performed according to the manufacturer's instructions.

### Statistical analysis

Significant differences (P < 0.05) between APP results of the infected and control groups for each day were determined by calculation of the approximate 95% interval for the difference in population means [28]. Area under the curve (AUC) was calculated using the trapezoidal rule (SigmaPlot version 8.02, Systat Software, London, UK). The significance of differences between AUC for infected and control groups determined by the Mann-Whitney test for non-parametric data using Minitab version 14 (Minitab Ltd Coventry, UK)

## Authors' contributions

PDE, WD and MCF originated and facilitated the study, with the former collating results and drafting the paper. FPL, LB and MW developed and performed the acute phase protein assays. MCF organised and supervised the animal studies and the IgG analyses which were performed by TLL. All authors have read and approved the final manuscript.
